# Trustwide Clinical Audit of Dementia Diagnosis: How Are Diagnoses Made and What Treatments Are Offered?

**DOI:** 10.1192/bjo.2025.10552

**Published:** 2025-06-20

**Authors:** Olusegun Sodiya, Oluwatosin Atewogboye, Roseline Ataria, Mani Krishnan, Rachael Raw

**Affiliations:** 1 Tees,Esk and Wear Valleys NHS Foundation Trust, Stockton, United Kingdom; 2 Cumbria, Northumberland, Tyne and Wear NHS Foundation Trust, Newcastle, United Kingdom

## Abstract

**Aims:** To assess variations in dementia treatment, diagnosis, and pathway practices across the memory services in Tees, Esk and Wear Valleys (TEWV) NHS Foundation Trust and how these compare to Trust and national policy. There were reports that the dementia diagnostic process across the trust was inconsistent. Thus, it was important to find out if the diagnostic pathway differed. NICE guidelines NG97 (2018) recommended neuroimaging, cognitive testing, and history to make diagnosis and the use of validated criteria to diagnose dementia subtypes. NG97 (2018) and TEWV Dementia Pathway suggested use of cholinesterase inhibitors to those with Alzheimer’s disease, mixed dementia, dementia with Lewy bodies and Parkinson’s disease dementia. These guidelines mentioned that memantine should be offered to those with severe Alzheimer’s, or those with moderate Alzheimer’s disease with a contraindication to cholinesterase inhibitors.

**Methods:** A simple random sampling of 150 patients seen at diagnostic appointments across the Trust 15 memory service between July 2023 and June 2024. 10 patients were selected from each memory service across the trust. The final numbers that fulfilled the inclusion criteria and analysed were 142. The audit of the collated data was done between July and September 2024. The analysis of the data, including inferences, deductions and diagrams was done using Excel.

**Results:** Across the trust, patients were given a dementia subtype where possible in 100% of cases. The overall practice in offering medications when indicated was 96% and 10 out of the 15 memory service achieved 100% in this area. There were clear documentations in most areas regarding the rationale for not prescribing medications or switching to another alternative. In the study, mixed dementia was the most common diagnosis by subtype, representing 35% of the data analysed, followed by Alzheimer’s dementia with 32% of the data analysed. Only 2 memory services offered Cognitive stimulation therapy (CST) to all the patients who were suitable. 10 teams scored below 40% in offering CST, and 3 teams scored between 50 and 67% for CST.

**Conclusion:** There was consistency across the Trust in the domain of considering clinical history to make a diagnosis. This good practice extends to the use of neuroimaging, cognitive screening and in some cases neuropsychology to make a diagnosis. A limitation of this study is that the small sample size per service could have limited the representativeness of the audit.

